# When Is Visual Information Used to Control Locomotion When Descending a Kerb?

**DOI:** 10.1371/journal.pone.0019079

**Published:** 2011-04-18

**Authors:** John G. Buckley, Matthew A. Timmis, Andy J. Scally, David B. Elliott

**Affiliations:** 1 School of Engineering, Design and Technology, University of Bradford, Bradford, West Yorkshire, United Kingdom; 2 Vision and Eye Research Unit, Anglia Ruskin University, Cambridge, Cambridgeshire, United Kingdom; 3 School of Health Studies, University of Bradford, Bradford, West Yorkshire, United Kingdom; 4 School of Optometry and Vision Science, University of Bradford, Bradford, West Yorkshire, United Kingdom; University of Sydney, Australia

## Abstract

**Background:**

Descending kerbs during locomotion involves the regulation of appropriate foot placement before the kerb-edge and foot clearance over it. It also involves the modulation of gait output to ensure the body-mass is safely and smoothly lowered to the new level. Previous research has shown that vision is used in such adaptive gait tasks for feedforward planning, with vision from the lower visual field (lvf) used for online updating. The present study determined when lvf information is used to control/update locomotion when stepping from a kerb.

**Methodology/Principal Findings:**

12 young adults stepped down a kerb during ongoing gait. Force sensitive resistors (attached to participants' feet) interfaced with an high-speed PDLC ‘smart glass’ sheet, allowed the lvf to be unpredictably occluded at either heel-contact of the penultimate or final step before the kerb-edge up to contact with the lower level. Analysis focussed on determining changes in foot placement distance before the kerb-edge, clearance over it, and in kinematic measures of the step down. Lvf occlusion from the instant of final step contact had no significant effect on any dependant variable (*p*>0.09). Occlusion of the lvf from the instant of penultimate step contact had a significant effect on foot clearance and on several kinematic measures, with findings consistent with participants becoming uncertain regarding relative horizontal location of the kerb-edge.

**Conclusion/Significance:**

These findings suggest concurrent feedback of the lower limb, kerb-edge, and/or floor area immediately in front/below the kerb is not used when stepping from a kerb during ongoing gait. Instead heel-clearance and pre-landing-kinematic parameters are determined/planned using lvf information acquired in the penultimate step during the approach to the kerb-edge, with information related to foot placement before the kerb-edge being the most salient.

## Introduction

Negotiation of an obstacle during gait requires an individual to determine the height and distance to the obstacle and plan appropriate foot placement and limb elevation for successful clearance. Gaze during the execution of such adaptive gait tasks is intermittently directed at the obstacle during the approach to it but the obstacle is not fixated during the step before or over the obstacle, and for the rest of the time gaze is directed on the ground ahead [1]. This highlights that vision is used in feed-forward planning and several studies have shown such planning determines both foot placement before and toe clearance over the obstacle [1]–[5].

Raised obstacles are not the only hazard we encounter during everyday locomotion. Like obstacle crossing, descending kerbs during ongoing gait involves the regulation of appropriate foot placement before the kerb-edge and foot clearance over it. However, unlike obstacle crossing it also involves the modulation of gait output to ensure the body-mass is safely and smoothly lowered to a new level. Indeed, most of us will have experienced stepping from a kerb we had not anticipated. The shock force generated travels up the leg to the base of the spine and is experienced as an uncomfortable ‘jolt’ to the lower back. Thus predicting at what height and hence when contact with the lower level occurs are critical factors [6]. These factors determine how and when the leading limb needs to be prepared for landing in order to safely and smoothly attenuate the increased downward momentum generated in lowering the body-mass to a new level; so that normal level walking can resume with minimal delay or perturbation. Vision has a predominant role in determining these critical factors [7]–[9], with vision from the lower visual field (lvf) being particularly important [10]. However, the insights regarding when and what visual information is used in the control of this adaptive gait task have been gained from work that has focussed on how landing control is regulated when step descents are completed from a stationary standing position [7]–[10]. In the present study we build upon this research and investigate how and when vision is used in the control of landing when stepping from a kerb during ongoing gait. Such research is important given that problems with stair negotiation and/or transitions between levels are common causes for falls [11]–[13], and that impairment of vision has consistently been cited as a contributing factor in falls [14]–[15].

As highlighted above, gaze during adaptive gait is directed two or more walking steps ahead [1]. This implies that when descending a kerb during ongoing gait, an individual is unlikely to look directly at their feet or the area on the ground they intend to step onto. Although gaze may be directed ahead, visual feedback of the lower-limb and/or floor area immediately in front/below the foot will be available from the lvf. Such feedback has been shown to be used online during obstacle crossing to update foot placement before the obstacle and toe clearance over it [3], [16]–[18], as well as detect the presence of an unexpectedly appearing obstacle [19]. In the above mentioned studies (except [19]), the importance of the lvf was highlighted by examining obstacle crossing with and without the lvf occluded. This was achieved by participants wearing goggles that occluded the lvf for the entirety of each walking trial. This meant the obstacle could be seen during the approach to it but not seen during the final step(s) before and step over the obstacle. Thus, although these studies were able to demonstrate the importance of lvf information to the online control of adaptive gait, they were unable to determine exactly when during the final 1 or 2 steps of the approach to the obstacle (or the step over it) such information is typically acquired/used. In the present study, we use a high-speed polymer dispersed liquid crystal (PDLC, ‘smart glass’) sheet (attached to the lower half of clear goggles) to unpredictably occlude vision from the lvf for certain periods during the final approach to, and step from a kerb. The aim of the study was to determine when vision from the lvf is used to control locomotion when stepping down from a kerb during ongoing gait.

## Materials and Methods

### Participants

12 healthy adults (6 male and 6 female), age 22±2.5 years (mean ± SD), height 175.7±8.5 cm and mass 68.2±8.1 kg, with no self-reported balance, gait or eye abnormalities volunteered to take part. The tenets of the Declaration of Helsinki were observed and the experiment gained approval from the University of Bradford's Committee for Ethics in Research. Written informed consent was obtained from each participant prior to undertaking the study. Binocular visual acuity, binocular contrast sensitivity and stereoacuity (depth perception) were each assessed using standard techniques (as described in [20]) and all individuals recorded values within the limits of healthy eyes [20], with measures of visual acuity, contrast sensitivity and stereoacuity of −0.23±0.06 logMAR (Snellen equivalent 6/3.5), 1.95±0.02 log units, and 39±14.5 secs of arc respectively.

### Protocol

**Figure 1:**
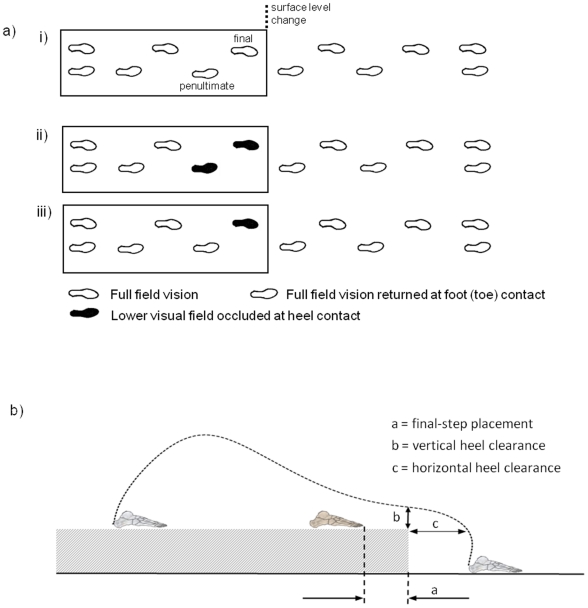
Schematic of the experimental set-up and how foot placement and heel clearance parameters were determined. a) The three visual conditions: i) no visual occlusion, ii) lower visual field occlusion from penultimate step heel contact iii) lower visual field occlusion from final step heel contact, and b) foot placement and heel clearance parameters.

Participants walked along a raised surface (14.6 cm high, 3 m long and 1 m wide) before stepping down onto the floor level and continued walking along the laboratory floor for at least 5 walking steps (see figure 1a). Start position was either 4 or 5 walking steps away from the surface (kerb) edge (randomly varied), and participants were required to negotiate the kerb leading with their right leg. Thus if participants started from 5 steps away they initiated gait with their right leg, and if they started from 4 steps away they initiated gait with their left leg. The variations in starting distance increased participants' reliance on using visual information when stepping from the kerb rather than simply adopting a repeated motor strategy. A force-platform mounted in the floor collected ground reaction force data (at 100 Hz) for the lead-foot contact onto the floor. The raised surface was constructed from plywood and covered in the same green vinyl as the surrounding floor. The laboratory was well lit with ambient illuminance of 400 lux measured at eye level.

Force sensitive resistors (FSR, Delysis, Boston, USA) were attached to the soles of each shoe, 1 cm anterior and 1 cm lateral of the midpoint of the shoe's posterior border (i.e. approximate location of point of contact during overground walking). An additional FSR was attached to the sole of the right shoe underneath the 2^nd^ metatarsal head (i.e. approximate location of point of contact when stepping down to a new level). Participants wore plastic goggles (Protector Safety, England) with a thin-flexible ‘smart glass’ sheet, incorporating a high-speed PDLC film, attached across the lower half of the goggles so that the sheet's upper edge was in line with the middle of the pupil. The response time of the PDLC sheet when switching from transparent to translucent or vice versa, was approximately 5 ms (determined experimentally [21]). With the sheet held in front of the eyes, the transparent state had no affect on vision, i.e. visual acuity and contrast sensitivity were unchanged. However, in the translucent state vision was degraded to a bare minimum, i.e. visual acuity was reduced to 1.5 logMAR (Snellen equivalent, 2/60), and contrast sensitivity was reduced to 0.15 log units. Signals from the FSRs were fed to a control box which was used to switch the PDLC sheet from transparent to translucent at either heel-contact of the penultimate (right) or final (left) step before the kerb-edge, and then from translucent to transparent at lead (right) foot (toe) contact with the lower floor level (figure 1a). Any trial that was not completed according to these instructions was discarded and repeated.

Lvf occlusion at instances of penultimate or final step contact were randomly presented with a 1∶5 ratio (occlusion: no occlusion). This low perturbation ratio ensured participants would not plan for ‘the worst case scenario’ ([22], i.e. that the lvf would be occluded) and therefore would not give greater weighting to central visual cues/feedforward mechanisms [23]. A number of ‘dummy trials’ were also completed to reduce the effectiveness of using somatosensory feedback from previous trials to predict the height of the lower level. These (which formed part of the full vision trials) involved increasing the height of the raised surface, out of view of participants, by 15 mm (to give a total height of 161 mm) and were undertaken every third trial. No data were collected during dummy trials and participants were advised that the height of the raised surface would be varied throughout the study. Lvf occlusion trials (from instant of either penultimate or final step contact) were repeated 3 times, so that participants completed a total of 36 trials (6 perturbed and 30 unperturbed).

Kinematic data were collected (at 100 Hz) using an 8 camera 3-D motion capture system (Vicon MX3, Oxford Metrics Ltd). Data were collected during a single testing session for each participant, with adequate rest periods provided to prevent fatigue. Participants wore their own shorts, t-shirt and flat-soled shoes they deemed comfortable for walking. Retro-reflective spherical markers (14 mm or 9 mm diameter) where attached either directly to the skin or clothing at key anatomical landmarks (as per Plug-In Gait guidelines; Vicon Oxford Metrics: see [10] for specific details). Markers were also placed on the kerb-edge to determine its location/height within the laboratory coordinate system. Using the Plug-In Gait software (Oxford metrics Ltd), marker trajectory data were filtered with the Woltring spline-smoothing routine with the mean square error (MSE) filter option set to 10, and then processed to define a 3-D linked-segment model of the participant incorporating anthropometric measurements taken of the participant.

### Data analysis

Analysis focussed on determining changes in kinematic variables that we and others have previously found important in crossing obstacles and descending steps [2]–[5], [7]–[10]. The variables we assessed were; final foot placement distance before the kerb-edge and heel clearance over it (figure 1b), minimum head flexion (pitch) angle at key points during the approach to and step from the kerb, kneedrop and time of kneedrop (see [8] for details regarding kneedrop) for the step descent movement, and the ankle angular displacement, peak vertical (downwards) body-mass velocity and peak vertical ground contact force during landing.

Head pitch angle was measured to check whether participants increased the amount of head flexion when the lvf was occluded in an attempt to receive visual information of the lower limb and/or surrounding floor area in their upper visual field. Local minima head-flexion angles were determined from penultimate step contact to final step contact to ground contact with the lower level.

Time of lead limb foot-off to ipsilateral foot contact with the lower floor level (swing duration), and foot contact to contra-lateral limb foot-off (weight transfer time) were also evaluated [24]. Lead- and trail- limb foot-off were defined as the instant the anterior/posterior velocity of each foot's toe marker first increased above 150 mm/s following the period of zero velocity when the foot was planted (penultimate and final foot placement respectively) on the raised surface. The duration of lvf occlusion in each condition, i.e. time from penultimate or final step contact up to ground contact with the lower level, was also determined.

### Statistical analysis

Data were analysed using random effects population averaged modelling techniques using the Stata version 8.0 statistical programme (Stat Corp., College Station, USA). This approach is an extension of the repeated-measures ANOVA approach (see [25]). This multivariate statistical model was obtained using the ‘xtreg’ command that uses the generalized least squares (GLS) random-effects estimator, to produce a matrix-weighted average of the between-subjects and within-subject output. The essential feature of such a model is that it takes into account that readings for a particular individual are likely to be correlated [25]. The model had just one specific term; vision with three levels: i) full vision available throughout, ii) lvf occlusion from instant of penultimate step contact, and iii) lvf occlusion from instant of final step contact. Significance of this three-level factor was determined using the ‘Z’-statistic. In essence this approach compared each lvf occlusion condition to the full vision available throughout condition. In all, 9 dependent variables were analysed in this way (with 11 separate statistical tests performed, including analysis of head-flexion angles at three different time points).

If any of the above Z-tests demonstrated statistical significance, a post-hoc analysis of the effects of lvf occlusion from instant of penultimate step contact compared to final step contact was determined using a likelihood ratio (*χ* ^2^) test, after first dropping ‘full vision available throughout’ from the model.

Due to the exploratory nature of the study no type I error adjustment of the alpha level was deemed necessary. Level of significance was thus set at *p*<0.05.

## Results

**Figure 2:**
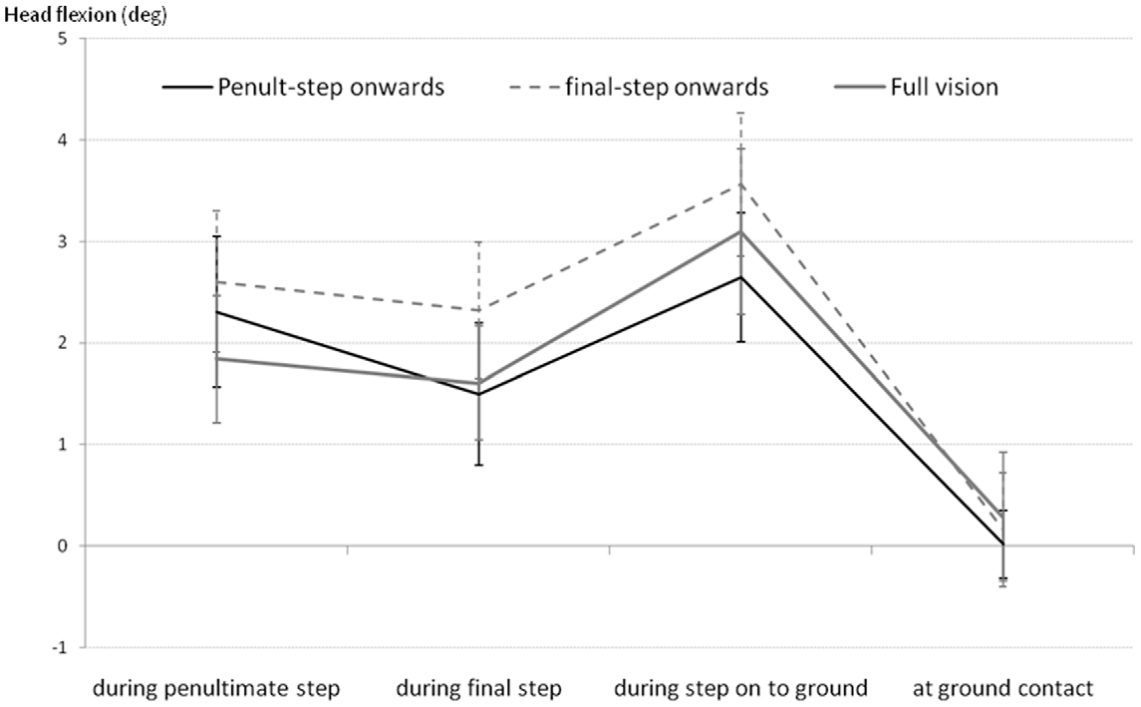
Group mean (±SE) peak amount of head-flexion (deg) occurring during the penultimate and final steps before the kerb-edge, step on to lower level and at instant of ground contact.

Lvf occlusion occurred unpredictably at the instants of penultimate step or final step contact up to ground contact with the lower level, and the resulting occlusion durations averaged 1.378 (±0.063) and 0.756 (±0.074) seconds respectively. Foot placement distance before the kerb-edge and peak ground contact force during landing were unaffected by vision condition (*p*>0.77, table 1). During the approach to and step from the kerb, local minima head-flexion angles were unaffected by lvf occlusion (*p* = 0.43), indicating there were no significant differences in the amount of head-flexion across the three vision conditions (figure 2). Moreover, lvf occlusion from the instant of final step contact up to the instant of ground contact with the lower floor level had no significant effect on any dependant variable (*p*>0.09). In contrast, occlusion of the lvf from the instant of penultimate step contact up to the instant of ground contact with the lower floor level had a significant effect on several kinematic measures (see following sections). The changes found have been shown in previous studies to relate to cautious behaviour [7]–[8], [10].

**Table 1:** Group mean (±1 SD) kinematic measures for the full vision and lvf occlusion conditions: a) measure occurring during approach, b) measures occurring during the step on to floor, and c) measures occurring during landing.

	lvf occlusion condition			
	No occlusion	Final step onwards	Penult step onwards	Interaction F v P
Final-step foot placement (mm)	140 (52)	139 (43)	137 (36)	
Heel-clearance_Hor_ (mm)	153 (60)	155 (55)	169 (71)*	0.091
Heel-clearance_Vert_ (mm)	53 (18)	54 (17)	61(21)∧	0.003
Kneedrop (mm)	97 (17)	97 (16)	92 (19)*	0.076
Time kneedrop (% swing duration)	85 (4)	85 (4)	84 (4)*	0.031
Ankle angular displacement (deg)	7.6 (6.4)	8.1 (6.1)	8.7 (5.8)*	0.50
Ground-contact force peak (N)	935 (171)	949 (183)	922 (163)	
Peak vertical CoM velocity (cm/s)	−445 (132)	−444 (135)	−464 (138)^t^	

Significant differences to full vision condition are shown by an asterisk * (*p*≤0.05) or ∧ (*p*≤0.001), and superscript ‘t’ indicates difference trend (*p* = 0.065). P-values in right-hand column are the result of the post-hoc analysis of differences between the two visual occlusion conditions.

**Figure 3:**
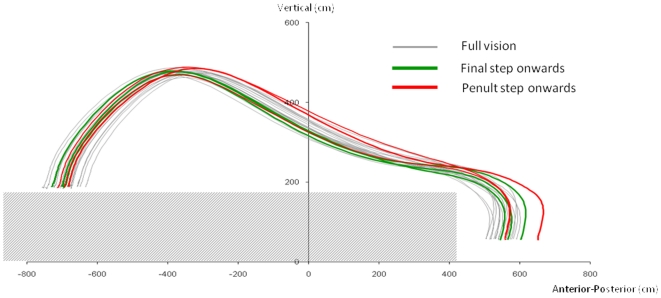
Lower visual field influence upon heel trajectory. Trajectories of the heel marker are shown for all trials from one subject for the step onto the lower floor level. Trajectories are plotted from lead-limb toe-off up to instant of contact with the lower level. NB, the heel trajectory for the condition when lvf was occluded at instant of penultimate step contact onwards, was beyond or towards the upper part (indicating higher heel clearance) of the variability boundaries of the full vision available throughout condition.

Lvf occlusion at the instant of penultimate step contact resulted in the following changes (relative to full field vision available throughout). Lead-foot mean vertical (z = 4.11, *p*<0.001) and horizontal (*z* = 2.70, *p* = 0.007) heel clearance (see figure 3) and ankle angular displacement (z = 2.17, *p* = 0.03) during landing became significantly increased, while kneedrop became significantly decreased (z = −2.16, *p* = 0.03), and timing of kneedrop occurred significantly earlier (*z* = −2.82, *p* = 0.005, table 1). There was also a tendency for these variables to be different from that recorded for when lvf was occluded at final step contact (table 1). However, only the differences in vertical heel clearance (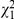 = 8.65, *p* = 0.003), and time of knee-drop (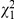 = 4.63, *p* = 0.031) reached levels of significance. Body-mass downward velocity during landing also became increased when lvf was occluded at instant of penultimate step contact, but the increase was only a trend (*p* = 0.065).

Weight transfer time was significantly increased (z = 2.50, *p* = 0.013) following lvf occlusion at instant of penultimate step contact, whereas swing duration was unaffected (*p* = 0.40, table 2). Weight transfer time when lvf was occluded at penultimate step contact was not significantly different to that when lvf was occluded at final step contact (*p*>0.22).

**Table 2:** Group mean (±1 SD) temporal parameters for the full vision and lvf occlusion conditions.

	Vision Condition		
	No occlusion	Final step onwards	Penult step onwards
Swing duration(s)	0.651(0.082)	0.654(0.093)	0.659(0.099)
Weight transfer time (s)	0.088(0.027)	0.091(0.026)	0.095*(0.035)

Significant difference to full vision condition is shown by an asterisk * (*p*≤0.05). Post-hoc analysis of the difference in weight transfer time between the two visual occlusion conditions indicated a non significant difference (*p* = 0.22).

## Discussion

The key findings of the present study were that, compared to full field vision available throughout, lvf occlusion from the instant of final step contact had no significant effect on any of the assessed variables, while lvf occlusion from the instant of penultimate step contact had a significant effect on several of the assessed variables.

Lvf was occluded by switching a PDLC sheet positioned with its upper edge in line with the pupils when in primary gaze, from transparent to translucent at the instants of penultimate step or final step contact up to ground contact with the lower level. Occlusion duration times for these two visual perturbation conditions were 1.378 (±0.063) and 0.756 (±0.074) seconds respectively. Once the sheet was switched to translucent the head would have needed to be flexed by 60 degrees or more in order to see the ground within 1 to 2 metres in front of the feet. Flexing the head by this amount would likely cause a significant perturbation to balance, and no participant had this amount of flexion, with average values around 2 degrees during the approach and step from the kerb-edge, and no significant difference in the amount of head-flexion across the vision conditions (see figure 2).

**Figure 4:**
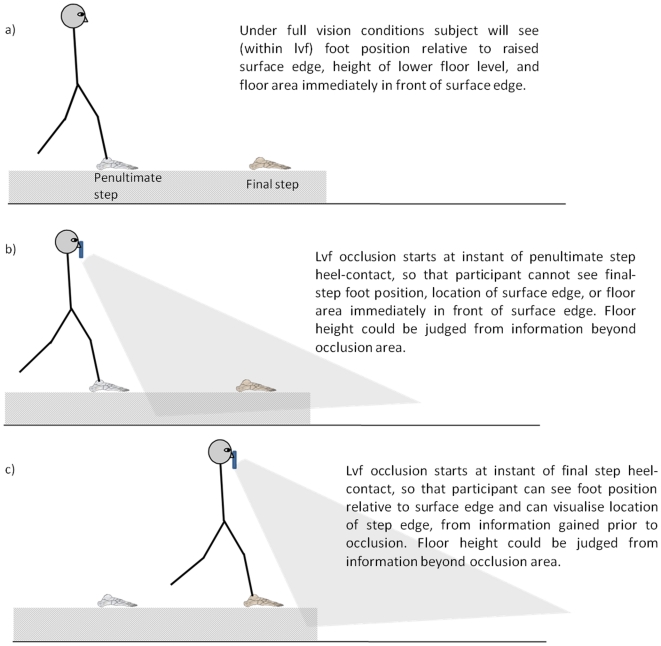
The three visual conditions.

In both lvf occlusion conditions participants would have been unable to see the kerb-edge or area on the floor they were stepping to during the step onto the lower floor level (see figure 4). The key difference between the two visual occlusion conditions is that participants obtained visual feedback regarding final-step foot placement relative to kerb-edge when the lvf was occluded at instant of final step contact (due to information gained during the swing phase immediately prior to the occlusion), but didn't obtain such feedback when the lvf was occluded at instant of penultimate step contact (figure 4). Thus participants would have been able to store a cognitive representation [26] regarding the relative position of kerb-edge during the step down with greater precision/certainty when lvf was occluded at final-step contact in comparison to when it was occluded at penultimate-step contact. The significant increase in mean vertical and horizontal heel clearance over the kerb-edge when lvf was occluded from penultimate step contact onwards, indicates participants were indeed uncertain (less precise) about the horizontal location of the kerb-edge and as a consequence increased margins of safety.

Previous research has highlighted that a key kinematic marker involved in step descent is the parameter kneedrop [8]. Kneedrop represents how far the knee has moved downwards, from its initial position, at the instant when the swinging (lead) lower-limb reaches its peak forwards swing before beginning to swing backwards. Under full undisrupted vision conditions kneedrop is scaled to the height of the surface level change [8]. In the present study kneedrop distance and timing of kneedrop were found to be significantly reduced when lvf was occluded at the instant of penultimate step contact (see table 1). This suggests that as well as being uncertain about the horizontal location of the kerb-edge, participants also became uncertain regarding precise floor height when stepping down if lvf information was occluded during the penultimate step before the kerb-edge. This was despite visual information regarding relative floor height being available in the upper visual field, i.e. from above the occlusion sheet (figure 4). The significant change in kneedrop distance and timing suggests participants prepared for landing earlier [10] when they were uncertain regarding precise height and location of the floor area they were stepping down to. We have previously highlighted that a decrease in kneedrop distance and timing, as found in the present study when lvf was occluded at penultimate step contact, results in an increased foot angle relative to the floor, so that landing occurs more ‘on the toes’ [10]. An increased foot angle would mean the heel needs to travel further vertically to attain a foot-flat position following ground-contact. Controlled lowering of the foot (and by implication the body-mass) is a way of attenuating the force of landing [27]–[28]. This change in landing behaviour explains why ankle angular displacement during landing and weight transfer time were both found to increase when lvf was occluded at penultimate step contact (and why there was also a trend [p = 0.065] of increased downward body-mass velocity during landing).

It is worth emphasising that although there was uncertainty regarding kerb-edge location and precise floor height when lvf was occluded from penultimate step contact onwards, there were no trips or stumbles, and no reduction in stepping distance; indicating there was no fundamental alteration in ongoing gait. This suggests that the primary planning of the step-down onto the floor level was undertaken using (full-field) visual information acquired during the approach to the kerb-edge. It also suggests that lvf information acquired between penultimate step and final step contact (when available) was used to update/confirm trail-limb (final step) foot placement before the kerb-edge; which in turn was used to determine/plan lead-limb foot swing trajectory over the kerb-edge and prior to landing. These findings are in agreement with previous work highlighting that the occlusion of the lvf prior to and during step execution when stepping down from a stationary standing position, caused significant changes in landing control but without fundamentally altering stepping strategy [10]. They are also in general agreement with previous work on adaptive gait involving obstacle negotiation, where occlusion of the lvf resulted in a lack of ‘fine-tuning’ of lower limb trajectory during point of crossing [3], [16]–[18].

In the above mentioned obstacle crossing studies, lvf was occluded by participants wearing goggles that obstructed vision for the entirety of each walking trial. As highlighted by the authors of these studies, this allowed the obstacle (that was to be crossed) to be seen during the approach to it but not seen during the final step(s) before or step over the obstacle. This means that the authors' interpretation, that the increases in foot placement distance from and toe clearance over the obstacle when the lvf was occluded, indicated that a view of the lower limb as it crossed the obstacle was an important factor in controlling lower limb trajectory, may need revising. According to the findings of the present study it is visual information from the lvf acquired between one and two walking steps in advance (of the obstacle) that is important: not concurrent lvf information. Indeed compared to full field vision available throughout, occlusion of the lvf from final step contact up to contact with the lower level had no significant effect on any dependent variable. This implies that visual feedback regarding trail-limb (final step) foot placement before the kerb-edge was the important information gained from the lvf. Future work is required to confirm whether this is also the case for other adaptive gait tasks.

When negotiating a floor-based obstacle during ongoing gait on a level surface the key requirement is to avoid contacting the obstacle. Thus it could be argued that this task will have greater reliance on using online lvf information because of the need to update both obstacle height and relative position information. In contrast, when stepping from a kerb the relative height of the lower level can be updated using information available in the upper visual field; and thus there is perhaps relatively less risk of tripping. However, previous research has shown that success rate in crossing an obstacle is not due to inappropriate limb elevation but is related to incorrect foot placement before the obstacle [29]. Such findings suggest, in agreement with the findings of the present study, that it is visual feedback regarding trail-limb (final step) placement prior to the obstacle that is paramount: but again future work is required to confirm this.

In summary, findings suggest that concurrent feedback of the lower limb, kerb-edge, and/or floor area immediately in front/below the kerb-edge, is not important when stepping from a kerb during ongoing gait. Instead foot-clearance and other key kinematic parameters of the stepping movement are determined/planned using lvf information acquired prior to final step contact, with information related to final-step foot placement before the kerb-edge being the most salient.
